# Bowel disorders in patients with severe acquired brain injury receiving neurorehabilitation: results from a national survey in Italy

**DOI:** 10.3389/fneur.2025.1474360

**Published:** 2025-06-02

**Authors:** Domenico Intiso, Andrea Montis, Federico Scarponi, Bahia Hakiki, Anna Cassio, Rossella Lopes, Susanna Lavezzi

**Affiliations:** ^1^Unit of Neuro-rehabilitation and Rehabilitation Medicine, IRCCS “Casa Sollievo della Sofferenza”, San Giovanni Rotondo, Foggia, Italy; ^2^Rehabilitation Medicine and Neurorehabilitation Unit, ASL, Oristano, Italy; ^3^Department of Rehabilitation, San Giovanni Battista Hospital, ASL 3, Foligno, Perugia, Italy; ^4^IRCCS Don Carlo Gnocchi ONLUS, Firenze, Italy; ^5^Department of Clinical and Experimental Medicine, University of Florence, Florence, Italy; ^6^Physical and Rehabilitation Medicine, AUSL Piacenza, Piacenza, Italy; ^7^Functional Recovery and Rehabilitation Unit, Cannizzaro Emergency Hospital, Catania, Italy; ^8^Severe Brain Injury Rehabilitation Unit, Department of Neuroscience and Rehabilitation, S. Anna University Hospital, Ferrara, Italy

**Keywords:** bowel disorders, severe acquired brain injury, rehabilitation, survey, management healthcare

## Abstract

**Background:**

Several studies have focused the relationship between cerebral lesions and gut function that has been defined as “brain-gut axis.” However, patients with brain damage can also present with different bowel dysfunctions (BDs), frequently observed during rehabilitation stay, such as fecal incontinence, constipation and diarrhea. Limited data has been reported in the literature about the occurrence and management of BDs and no study has yet investigated this issue in patients with severe acquired brain injury (sABI).

**Aim:**

To investigate basal appraisal regarding BDs issue and therapeutic management in patients with sABI during rehabilitation; interest in examining the issue in depth and improving the knowledge of BDs.

**Design:**

Cross-sectional.

**Setting and population:**

Physiatrists or neurologists, 99 in total, working in 33 Italian rehabilitation centers involved in the care of sABI giving a pooled sample of 1835 inpatients.

**Method:**

On behalf of the SIMFER a **s**urvey questionnaire consisting of 19 close-ended questions with multiple-choice answers and 1 open-ended question was drafted. Items regarded the respondents’ opinions and experiences regarding the epidemiology and management aspects of BDs in patients with sABI. Survey data were collected anonymously via e-mail between July and October 2023.

**Results:**

All participants knew BDs, but only 18 (40%) and 19 (42.2%) participants were able to provide a definition of diarrhea and constipation, respectively. Eighteen (40%) participants used specific measurements to evaluate BDs. Variable laboratory and instrumental ascertainments were used in diagnosing diarrhea and 84.4% participants requested specialized consultation. All respondents answered that modality and type of nutrition were important in managing BDs. Thirty-six (80%) respondents considered that BDs might increase the incidence of urinary infections and 53.3% answered that BDs might negatively influence the ventricular peritoneal shunt. Participants (95.5%) agreed that mobilization could improve BDs and considered that BDs might hamper recovery (84.4%). The majority showed interest in increasing their knowledge on the topic.

**Conclusion:**

The survey detected limited knowledge and uncertainty about epidemiology, definition, and management of BDs in sABI patients, but also interest in exploring and educating oneself on the issue. Future studies should be planned to investigate the burden and the therapeutic strategy to manage BDs.

## Introduction

Patients with brain damage can commonly complain of gut dysfunctions. In the last few decades, an increasing number of studies has focused on the comorbidity between cerebral lesions and gut function, particularly in patients with Traumatic Brain Injury (TBI) (1, 2). The relationship between brain and gut has been defined as “brain-gut axis.” In this regard, the brain can regulate intestinal function such as gut motility, intestinal barrier, and visceral pain. Brain injuries may be related to (a) gastrointestinal disorders including gut mucosal alteration, increased permeability, inflammation, ulceration, and perforation; (b) motility disorders due to autonomic nervous system dysfunction; and (c) immune system activation (3). The effects of gut dysfunction on brain injury recovery are equally as important. For example, the microbiota is an important channel for information exchange between the intestine and the brain (4). Recently, several studies using animal models have demonstrated the role of the microbiota in regulating brain function and facilitating recovery after brain injury (5–8). Microbiota changes can affect both the acute and the chronic phase of recovery after cerebral damage particularly in patients with TBI (9–11) and can represent an interesting field of research for new approaches and therapeutic strategies.

However, patients with brain damage can also present with different bowel dysfunctions (BDs), frequently observed during rehabilitation stay, such as fecal incontinence, constipation and diarrhea. The burden and epidemiology of these disorders are not well known and limited to some diseases characterized by severe brain lesions, such as TBI and, more commonly, stroke. In stroke patients, the prevalence of fecal incontinence during rehabilitation stay has been reported in a range from 23 to 56%, that decreases at 11 to 21% after 3 months or at discharge (12). Likewise, constipation has been well documented. A recent systematic review showed that the incidence of constipation in stroke patients was 48% (95% CI: 33–63%) (13). On the other hand, there are very few epidemiological data on BDs in patients with TBI. A global incidence of 70–75% has been reported, with constipation being present in 32% of cases (14). Diarrhea is also a troublesome bowel disorder that is commonly observed in patients with cerebral damage receiving rehabilitation. However, no data has been reported for patients with TBI. The majority of TBI patients admitted in rehabilitation are coming from the Intensive Care Unit (ICU), therefore the occurrence of diarrhea during ICU stay could be considered as proxy data for these patients. In fact, an incidence of 69.6% has been detected in ICU patients with TBI, and those with diarrhea had a longer stay in the ICU (*p* = 0.007) (15). Several types of severe acquired cerebral injury (sABI) that produce disorder of consciousness (DOC), motor and sensorial impairments can require ICU admission and management. SABI is related to several different causes, including TBI, hypoxic brain injury, stroke, encephalitis and brain tumor. Patients with sABI can suffer from cognitive, communicative, physical, emotional, and behavioral limitations (16, 17) that require intensive rehabilitation.

Although frequently observed in clinical practice, no data is reported in the literature about the occurrence and management of BDs and no study has yet investigated the occurrence and the management of BDs in patients with sABI during rehabilitation stay. Guidelines and Consensus did not report specific recommendations regarding the management of BDs in patients with TBI. With this respect, the National Institute for Health and Excellence (18) reported generic indications, such as “patients should have a regular bowel regimen to avoid constipation and to manage fecal incontinence.” Scottish Guidelines (19) reported that “it is not possible based on the evidence reviewed to make a specific recommendation for treatment of incontinence in patients with brain injuries.” Only Canadian Guidelines (20) reported some therapeutic indications regarding constipation in TBI patients, such as including sufficient fluid intake, the use of natural laxatives, exercise and standing, privacy and comfort during defecation, and regular time of defecation each day. Likewise, the 3^th^ Italian National Consensus: good clinical practice guidelines for the rehabilitation of subjects with severe acquired brain injury did not consider the issue of BDs essential, attributing the disturbances to a disorder of consciousness or sensory-motor limitations, but not due to specific cerebral damage (21). The acknowledge and the management of BD by physicians is not investigated in Italy.

### Aims of the present study

Given the limitations of literature data and the lack of investigation, we performed a national survey of clinicians in Italy to investigate the following points: (1) basal appraisal regarding BDs issue in patients with sABI during rehabilitation; (2) therapeutic approach and management of BDs; (3) interest in examining the issue in depth and improving the knowledge of BDs of clinicians involved in the care of patients with sABI. The study was an initiative of the Direction Group of the section dedicated to sABI of the Italian Society of Physical Medicine and Rehabilitation (SIMFER).

## Methods

### Participants

The Italian National Health System recommends a specific care pathway for patients with sABI from the acute phase to the chronic condition. Consequently, each Regional Health System must implement this care pathway on their territory. In this respect, post-acute phase rehabilitation of patients with sABI is delivered in dedicated centers that are established and recognized by each Health Regional System. The length of stay (LOS) in these rehabilitation settings is related to the achievement of recovery or regardless of the functional outcome up to 6–12 months, particularly in patients with DOC.

On behalf of the SIMFER, a questionnaire was drafted by three senior experts (S.L.; D.I. and A.M.) in the care and rehabilitation of sABI, all members of the Direction Group on sABI and DOC (sABI&DOC), a national committee which is part of the sABI section of the SIMFER. To evaluate the feasibility and consistency of the items, the questionnaire was sent to senior physiatrists not involved in questionnaire processing. A revision was performed according to their comments and suggestions, and after collegial discussion and approval by the sABI&DOC Direction Group members, the final version of the questionnaire was sent by e-mail to all clinicians registered to the sABI&DOC section of the SIMFER.

**Figure 1:**
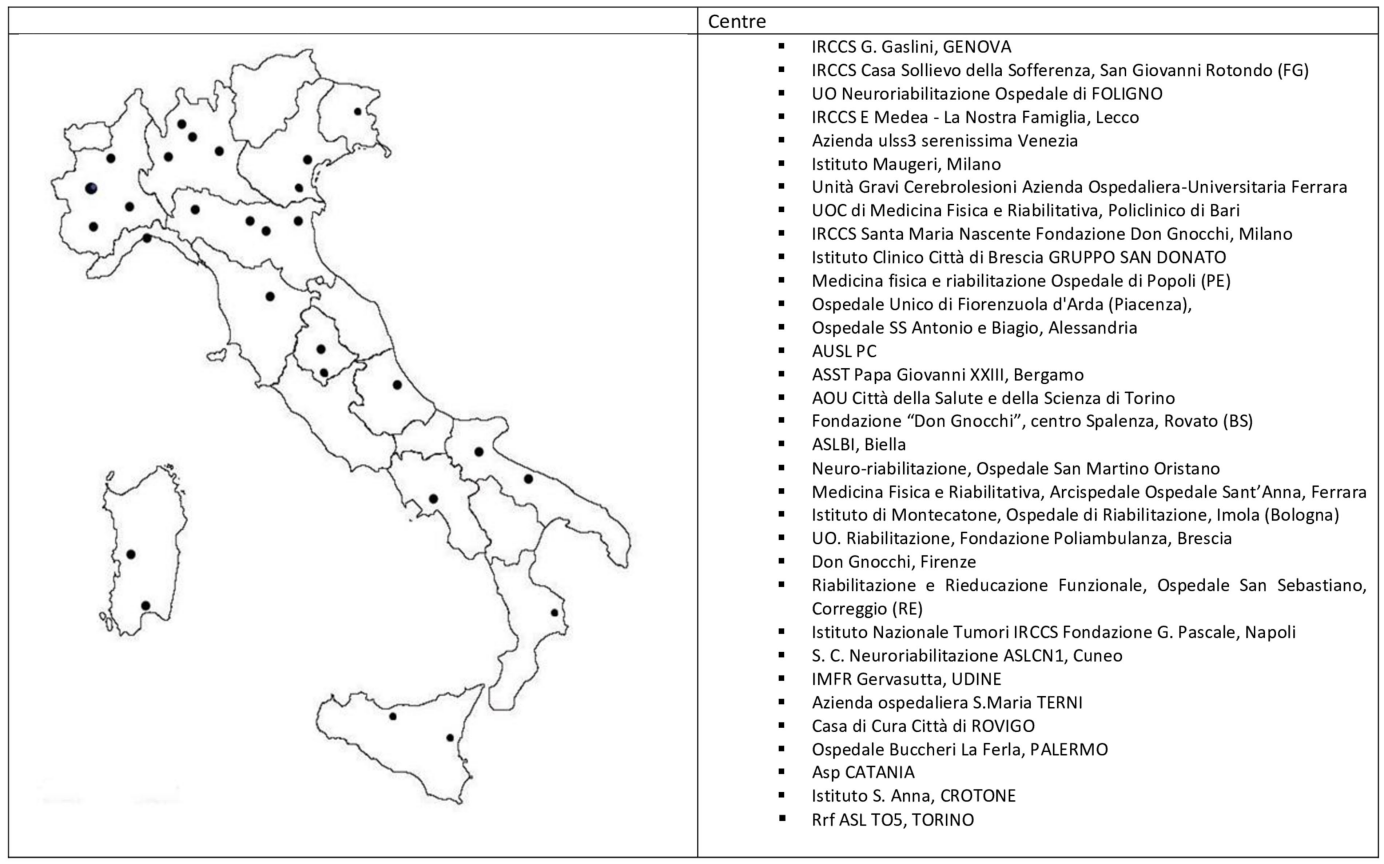
Site of centers admitting sABI patients.

Ninety-nine clinicians were registered to the sABI&DOC section of the SIMFER at the time of the survey and were sent the questionnaire via email. Of these, 45 (45.5%; *n* = 17 men, *n* = 28 women; mean age 47.7 ± 10.2) filled out and returned the questionnaire, and were hence included as participants in the present study. The respondents worked in 33 dedicated centers located in 28 cities, representative of almost all the national territory (see Figure 1). All clinicians who took part in the survey had at least one specialty and the majority (91.1%) were Physical Medicine and Rehabilitation specialists. The inclusion criteria for completing the questionnaire was being a neurorehabilitation physician (physiatrist or neurologist) working in a neurorehabilitation ward admitting adult inpatients with sABI in the post-acute phase. The survey was performed between July and October 2023. Participation was voluntary and all responses were confidential. No incentives was offered to provide the survey. Respondents were able to review and change their answers through a Back button. Each center provided only anonymous and aggregated data within the study, with no direct patient interview. All data were collected by the secretariat of the SIMFER. BDs were defined as clinical conditions that included the following intestine disorders: fecal incontinence, constipation and diarrhea. Since none of the clinicians who took part had a specific interest in BDs or was an expert in this field, constipation and diarrhea were defined according to the Bristol Stool Form Scale (BSFT) that works well in the clinic to assess consistency and correlates roughly with intestinal transit times. The BSFS is a scale that categorizes stools in seven types from the hardest to the softest. Types 1 and 2 to be uncharacteristically hard and indicative of constipation, while types 6 and 7 are unusually loose and indicate diarrhea (22). The BSFS has been demonstrated substantial validity and reliability (23). The present study was conducted according to the principles outlined in the Helsinki Declaration.

### Materials

The development of the questionnaire was based on the following criteria: item content had to (a) explore the knowledge and management of BDs in sABI; (b) be appropriate to the expected level of clinical knowledge and skills of the respondents; (c) explore the opinion of participants on the potential effects of BDs on the particular clinical conditions and patient outcomes. To ensure quality and reliability, the development process followed the CHERRIES checklist (24).

The questionnaire was composed of three sections. The first part concerned the rationale and aim of the study. A second section investigated the demographic characteristics of the clinician who filled out the questionnaire including specialty, institution, site of working center, and number of patients with sABI admitted yearly. The third and last part was composed of 20 items, including 19 close-ended questions with multiple-choice answers (Item 2–20), 4 of which included an open-ended part, and 1 open-ended question (Item 1). Given the lack of literature data, answers were facilitated by providing an even number of response categories without a middle response option stating a “neutral” response. In this regard, responses included percentages, such as “> 50%” or “< 50%,” or “YES” or “NO.” A further option was possible: “I do not know.” As mentioned, four of the close-ended questions included an open-ended part which concerned the management of diarrhea and constipation, in particular laboratory exams (Item 9) and therapeutic strategies (Item 10) employed for these disorders, as well as reasons of BDs effects on patient outcomes (Item 19).

Items 1–4 and 7–12 measured the knowledge of the participants about BDs (See Table 1A). In particular, Item 1 addressed the definition of fecal incontinence, constipation and diarrhea. Items 2–3 addressed the occurrence of BDs in patients with sABI (Item 2) or with sABI and DOC (Item 3) and the occurrence of fecal incontinence (Item 4) according to the participants. Items 7–11 assessed the measurements employed to evaluate BDs as well as the exams used to investigate the causes of the constipation and diarrhea (Items 8–9), and the therapy employed, including pharmacological (Item 10) or non-pharmacological (tools, devices) treatment (Item 11). In order to facilitate responding to Items 7–9 and 11, pre-indicated measures were used as possible responses. The respondents had to indicate what assessment they employed to evaluate BDs among the following: Wexner for constipation, Wexner for incontinence, Bristol Stool score, NBD score, Barthel Index, FIM, other (Item 7). Participants also indicated what exams they carried out when diagnosing constipation among the following: gastroscopy, colonoscopy, direct abdominal X-Ray, abdominal duplex scan, other (Item 8). Likewise, for Item 11, which addressed the use of non-pharmacological devices to manage BDs, the following instruments or tools were suggested: anal-plug, flex-seal, trans-anal irrigation, enema, other. On the other hand, Items 9–10, which assessed the examinations employed to diagnose diarrhea (Item 9), and type of treatments administrated to manage diarrhea and constipation (Item 10), were open-ended and there were no indications and the participants reported with their own diagnostic and therapeutic strategies.

**Table 1A:** Questionnaire (items 1–6): definition, epidemiology and nutrition modality of patients with sABI in the opinion of participants in the survey.

	≤50%	>50%	I do not know
	N°	N°	N°
*Item 1: What do you intend for bowel dysfunctions?			
Item 2: in your experience what is the percentage of sABI with BD?	16	24	5
Item 3: in your experience what is the percentage of sABI and DOC with BD?	1	41	3
Item 4: in your experience what is the percentage of sABI with fecal incontinence?	18	22	5
Item 5: in your center what is the percentage of sABI with nutrition by NGT, at entry?	23	21	1
Item 6: in your center what is the percentage of sABI with nutrition by PEG, at entry?	27	17	1

*Free response; BD, bowel dysfunctions; sABI, severe acquired brain injury; DOC, disorder of consciousness; NGT, nasal gastric tube; PEG, percutaneous endoscopy gastrostomy.

Items 5–6 and 12–15 assessed the relevance of nutrition and treatment interventions according to the respondents, and if specialized consultation and type was requested. As with previous items, participants had to choose between the following indicated strategies in case of constipation (Item 13) and diarrhea (Item 14): modification of velocity, time and frequency of infusion, modification of type of nutrition, probiotics, and food supplements.

Items 16–18 addressed what was the consideration of the respondents regarding the comorbidity between BDs and other clinical conditions, such as the effect of BDs on higher incidence of urinary infections (Item 16) and dysfunction of the ventricular peritoneal shunt (VPS; Item 17), as well as the role of mobilization to improve BDs (Item 18).

Furthermore, Item 19 addressed the potential effect of BDs on the functional outcome of patients with sABI. The last item concerned the interest of the respondent in receiving education and examining the issue of BDs in patients with sABI more in depth (Item 20; see Table 1B).

**Table 1B:** Questionnaire (items 7–20): management of BDs (constipation, diarrhea), BDs and nutrition, effects of bowel dysfunctions and interest for education.

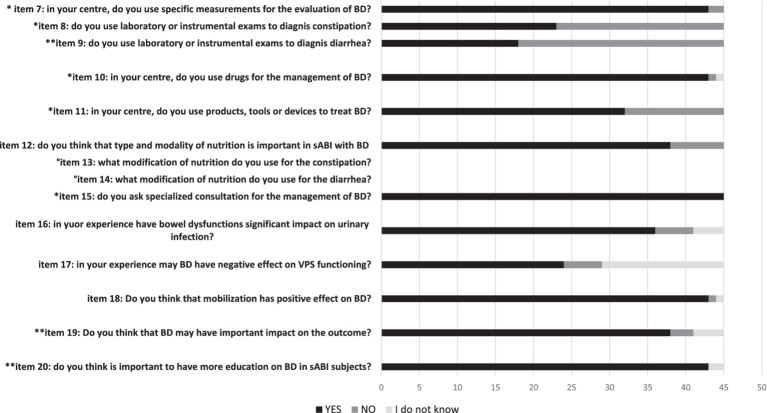

*In case of YES: list of indicated tools; **in case of YES: free response; °list of indicated strategies. BDs, bowel dysfunctions; sABI, severe acquired brain injury; VPS, ventricular-peritoneal shunt.

### Data analysis

The following analyses were performed:

- descriptive statistics for the epidemiological survey: respondents. We described the respondents using simple frequencies, characterizing their geographical distribution, age and sex.
- descriptive statistics for the opinion survey. We calculated simple frequency distributions of each response for each question.

## Results

All respondents completed questionnaires and no questionnaires was terminated early. Mean number of patients with sABI admitted yearly in dedicated neuro-rehabilitation settings was 61.3 (min 15, max 200) for a pooled sample of 1835 in-patients.

### Bowel dysfunctions: incontinence, constipation and diarrhea

**Figure 2:**
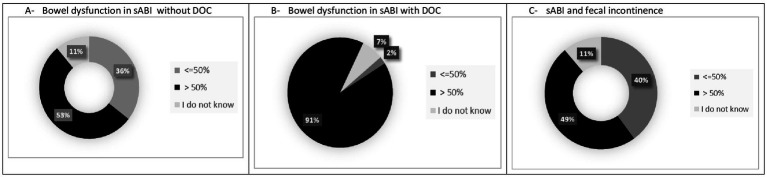
Occurrence of BDs in patients with sABI during neurorehabilitation stay in the opinion of participants in the survey. **(A)** Bowel dysfunction in sABI without DOC. **(B)** Bowel dysfunction in sABI with DOC. **(C)** sABI and fecal incontinence.

All participants knew that BDs included fecal incontinence, constipation and diarrhea, but only 18 (40%) and 19 (42.2%) of participants were able to provide a definition of diarrhea and constipation, respectively, according to BSFT. Twenty-four (54.5%) of participants answered that patients with sABI had a frequency of bowel dysfunction >50%, whereas the majority of respondents (91.1%) reported that sABI with DOC had >50% of bowel dysfunctions (see Table 1A; Figures 2A–C). However, only 18 (40%) of participants used specific measurements to evaluate BDs (see Table 1B). In particular, the most used measures were the Bristol Stool Score (*n* = 18); Barthel Index (*n* = 15) and FIM (*n* = 11). Twenty-three (51.1%) participants evaluated constipation by the use of variable instruments including abdominal duplex scan (*n* = 16), direct abdominal X-Ray (*n* = 21), gastroscopy (*n* = 4), colonoscopy (*n* = 6) and biopsy (*n* = 3). On the other hand, in case of diarrhea the majority of participants (95.5%) requested laboratory and instrumental ascertainments. Among these, exams included complete blood exams, feces exams, search of *Clostridium difficile* and instrumental assessments. Feces exams (*n* = 35) and search of *Clostridium difficile* (*n* = 35) were the most requested examinations.

### Bowel dysfunctions and nutrition

Item 12 addressed the role of nutrition in BDs according to the participants. sABI patients admitted in neurorehabilitation frequently suffer from severe dysphagia that requires devices such as the nasal gastric tube (NGT) or a percutaneous endoscopic gastrostomy (PEG). Twenty-one (46.6%) and 17 (37.7%) of respondents reported that a rate >50% of admitted patients with sABI needed the NGT and a PEG, respectively. All participants in the survey (100%) answered that modality and type of nutrition were important in managing BDs in patients with sABI. The majority agreed that it was important to modify the nutrition either in the case of constipation or diarrhea. Furthermore, the majority of respondents varied velocity of infusion (82.2%) and used probiotics (88.8%) to improve diarrhea. On the other hand, only around half of the sample varied velocity (51.1%) and used probiotics (66.6%) in case of constipation. The majority of respondents (84.4%) requested specialized consultation in particular by a nutritionist (*n* = 35), gastroenterologist (*n* = 20), infective specialist (*n* = 20), internist (*n* = 11) in the case of persistent BDs and to modify nutrition.

### Effects of bowel dysfunctions

Urinary tract infections can frequently occur in patients with sABI. Thirty-six (80%) respondents considered that BDs might increase the incidence of urinary infections. Many patients with sABI can have a VPS that in some circumstances may present a dysfunction due to several causes. 53.3% of the participants answered that BDs might negatively influence the VPS. The majority of participants agreed that mobilization could improve BDs (95.5%) and considered that BDs might hamper recovery (84.4%; Table 1B).

### Interest and education for BDs

All participants (95.5%) apart from 2 considered it useful to learn more about BDs and showed interest in increasing their knowledge on the topic.

## Discussion

Epidemiological data on the occurrence of BDs and its management in patients with sABI during rehabilitation stay are limited. We reported the results of a national survey launched by the SIMFER sABI&DOC section, which aimed to explore the appraisal of physiatrists involved in the care and rehabilitation of patients with sABI across 33 specialized centers in Italy. The survey detected limited knowledge and uncertainty about epidemiology, definition, and management of BDs, but also interest in exploring and getting educated on the issue. About the epidemiology of BDs, 54.5% of the sample reported that BDs occurred in >50% of sABI cases, whereas less than half (48.8%) of the participants responded that fecal incontinence had a rate of >50%. Furthermore, 11.1% of the participants reported that they did not know what was the possible rate of BDs. In this regard, although literature data are limited, a rate of 70–75% of global BDs has been reported in patients with TBI during rehabilitation (14). Fecal incontinence was detected in 68% of a sample of 1,013 consecutively enrolled rehabilitation inpatients with TBI, at admission (25). The disturbance improved during the rehabilitation stay and 12.4% still showed the disturbance at discharge. All participants knew that BDs included fecal incontinence, constipation and diarrhea, but only 40% of respondents defined BDs according to BSFT. Likewise, less than half of the sample used measures to evaluate BDs and in case of constipation, only 51.1% ascertained the cause by employing instrumental evaluations. Conversely, the majority of participants (95.5%) assessed the origin of diarrhea by using variable laboratory and instrumental ascertainments. This was an open-ended question and participants had to report what exams they employed to diagnose the origin of the diarrhea and the proposed exams based on the level of importance. A lot of examinations were reported including complete blood exams, feces exams, and in particular the search of *Clostridium difficile*. This aspect is understandable since hospital-acquired infections (HAIs) due particularly to multi-drug resistant agents are a serious health problem that impact on the rehabilitation pathway of patients with sABI, determining extended LOS, a significantly higher mortality and poorer functional outcomes (26). Among HAIs, *Clostridium difficile* infections are fairly common. The incidence of *Clostridium difficile* diarrhea is increasing and, since 2004, new types and outbreaks have emerged worldwide that are associated with higher morbidity and mortality (27). However, the present survey found that a wide variety of laboratory and instrumental exams were employed, showing the lack of standardized diagnostic pathways and procedures. Several products were used to treat constipation, in particular osmotic laxatives, prokinetics, stimulants (bisacodyl and sennosides), and fiber supplements. Likewise, a variety of drugs were used to treat diarrhea, including loperamide, probiotics, antibiotics and lactic enzymes.

The new insight and knowledge of the brain-gut axis opens a field of promising therapeutic approaches and strategies in patients with brain damage in which nutrition is an important aspect (28, 29). A lot of studies have been published about the role and the effect of nutrition in patients with TBI (30), but many questions remain unclear and debated such as nutritional status, the modality of nutrition (31, 32) and nutritional support (33). Some of the major risks are malnutrition and undernutrition (31, 34). The majority of patients with sABI admitted in neurorehabilitation receive enteral nutrition by the NGT or a PEG. Indeed, 51.1 and 60% of the sample responded that a rate >50% of sABI patients had nutrition by NGT and PEG, respectively, at entry. Whether nutrition type affects gut disorder is unclear. However, all participants agreed that type and modality of nutrition were important factors in patients with sABI who suffered from BDs. In this respect, therapeutic strategies including modification of food products, time and velocity of infusion, use of supplements and probiotics were used to treat either diarrhea or constipation. Furthermore, the majority of participants (84.4%) in the survey requested specialized consultation including from a nutritionist, gastroenterologist, infective specialist or internist, but the consultation was requested only in the case of persistent gut disorders, nutritional modification and suspected infection.

It is well known that patients with sABI receiving neurorehabilitation show clinical complexity with a huge of complications and conditions (35, 36). It would be interesting to know if BDs can be only one of the multiform clinical complications that may occur in patients with sABI and if potential or mutual relationships exist. In this respect, the present survey addressed urinary tract infections and VPS functioning that are frequent comorbid conditions in patients with sABI. VPS is performed in treating hydrocephalus and its functioning permits normal cerebral fluid drainage. Shunt placement is frequently associated with the need for later revisions as well as surgical complications and a rate of revisions is generally reported to be around 20–30% in the adult populations (37). VPS dysfunction and infection are the most frequent complications (38). In the present survey, 80% of respondents considered that BDs might have an impact on urinary infections, but the respondents were unclear if BDs might cause or favor it. Different opinions were reported about the relationship between BDs and VPS functioning. Only half (53.3%) of the sample agreed that BDs might negatively impact VPS functioning, and 35.5% answered “I do not know.”

Some clinical and neurological conditions and complications such as HAI (26), heterotopic calcification (39), paroxysmal sympathetic vegetative hyperactivity (40), critical illness polyneuropathy and myopathy (41, 42) occurring during rehabilitation stay in patients with sABI can hamper and limit functional outcomes. If BDs represent a clinical condition that may affect the recovery and produce functional limitations is unclear. In the present survey, a high percentage (84.4%) of the respondents considered that BDs might significantly affect functional outcomes of patients with sABI. This was an open-ended question and several reasons were mentioned including increase of complications (infections, pressure sores, metabolic and hydro-electrolytes alterations), worsening of nutritional status, a major caregiver burden, reduced rehabilitation interventions, and obstacles to functional recovery. On the other hand, unsurprisingly, the sample considered that mobilization could benefit BDs. Indeed, mobilization is one of the most important rehabilitation interventions, since early conventional and robotic-assisted mobilization has been proposed also in ICU patients (43, 44).

As previous mentioned, literature data on BDs in patients with sABI is limited and proxy published Guidelines such as those on TBI did not report specific recommendations about management of BDs. Only Canadian Guidelines report some therapeutic indications in the case of constipation including sufficient fluid intake, the use of natural laxatives, exercise and standing, privacy and comfort during defecation, and regular time of defecation each day. However, many of them do not mention rehabilitation setting and might not be applicable to patients with sABI. Importantly, the vast majority of participants (95.5%) considered it useful to obtain more information and education about BDs through proper events addressing the various aspects of gut disorders. Likewise, they consider it important to plan meeting involving multidisciplinary discussants, in order to individuate standardized therapeutic care pathways.

## Limitations of the study and future prospective

This study has several limitations. The survey reports clinicians’ opinions rather than data, which are generally collected by prospective observational studies. Clinicians who responded to the survey were not expert in the treatment of intestinal disorders, and did not have any specific training to manage it. Secondary, it is difficult to ascertain the extent to which our sample can be considered representative of all clinicians, even if the most of participants worked in rehabilitation centers dedicated to the care of patients with sABI. Many items had close-ended multiple-choice answers and the indicated responses could bias participants. The posed questions did not exhaustively explore the role of BDs, in particular the relationship between BDs and the varied clinical complexity of patients with sABI. Despite its limitations, the present study provides a picture of clinicians’ conduct in the management of BDs in patients with sABI during rehabilitation stay. Given the results, the sABI&DOC section of the SIMFER plans to organize educational events on the topic and to run an observational prospective study to better understand the burden and the management of BDs in patients with sABI. In addition, further education efforts should be made to improve the situation, such as holding meetings, organizing interdisciplinary experts to develop evidence-based practice guidelines.

## Conclusion

A national survey launched by the sABI&DOC section of the SIMFER explored physiatrists’ appraisal of BDs in patients with sABI receiving neurorehabilitation. The survey detected limited knowledge and uncertainness about epidemiology, definition, and management of BDs, but also interest in exploring and educating oneself on the issue. Future studies should be planned to investigate the burden and the therapeutic strategy to manage BDs, in order to design standardized care pathways.

## Clinical rehabilitation impact

Although frequently observed in clinical practice, no data has been reported in the literature on the occurrence and management of BDs in patients with sABI during rehabilitation stay and Guidelines and Consensus did not report recommendations. The present study provided a picture of clinicians’ conduct in the management of BDs in patients with sABI during rehabilitation stay and showed limited knowledge and uncertainty about epidemiology, definition, and management of BDs. It can be the basis to plan future studies to investigate the burden and the therapeutic strategies to manage BDs in sABI patients, in order to design standardized care pathways.

## Data Availability

The raw data supporting the conclusions of this article will be made available by the authors, without undue reservation.
